# MDM2 antagonist Nutlin-3a potentiates antitumour activity of cytotoxic drugs in sarcoma cell lines

**DOI:** 10.1186/1471-2407-11-211

**Published:** 2011-05-30

**Authors:** Hege O Ohnstad, Erik B Paulsen, Paul Noordhuis, Marianne Berg, Ragnhild A Lothe, Lyubomir T Vassilev, Ola Myklebost

**Affiliations:** 1 Department of Tumour Biology, The Norwegian Radium Hospital, Oslo University Hospital, P O Box 4953 Nydalen, NO-0424 Oslo, Norway; 2 Department of Cancer Prevention, The Norwegian Radium Hospital, Oslo University Hospital, P O Box 4953 Nydalen, NO-0424 Oslo, Norway; 3 Center for Cancer Biomedicine, University of Oslo, 0316 Oslo, Norway; 4 Roche Research Center, Hoffmann-La Roche Inc., Nutley, NJ 07110, USA; 5 Institute for Molecular Bioscience, University of Oslo, 0316 Oslo, Norway

## Abstract

**Background:**

Frequent failure and severe side effects of current sarcoma therapy warrants new therapeutic approaches. The small-molecule MDM2 antagonist Nutlin-3a activates the p53 pathway and efficiently induces apoptosis in tumours with amplified *MDM2 *gene and overexpression of MDM2 protein. However, the majority of human sarcomas have normal level of MDM2 and the therapeutic potential of MDM2 antagonists in this group is still unclear. We have investigated if Nutlin-3a could be employed to augment the response to traditional therapy and/or reduce the genotoxic burden of chemotherapy.

**Methods:**

A panel of sarcoma cell lines with different *TP53 *and *MDM2 *status were treated with Nutlin-3a combined with Doxorubicin, Methotrexate or Cisplatin, and their combination index determined.

**Results:**

Clear synergism was observed when Doxorubicin and Nutlin-3a were combined in cell lines with wild-type *TP53 *and amplified *MDM2*, or with Methotrexate in both *MDM2 *normal and amplified sarcoma cell lines, allowing for up to tenfold reduction of cytotoxic drug dose. Interestingly, Nutlin-3a seemed to potentiate the effect of classical drugs as Doxorubicin and Cisplatin in cell lines with mutated *TP53*, but inhibited the effect of Methotrexate.

**Conclusion:**

The use of Nutlin in combination with classical sarcoma chemotherapy shows promising preclinical potential, but since clear biomarkers are still lacking, clinical trials should be followed up with detailed tumour profiling.

## Background

The *TP53 *gene, coding for the transcription factor p53, is thought to be the most frequently mutated gene in cancer, inactivated in about 50% of all tumours. However, aberrations of this pathway are probably even more wide-spread, as tumours retaining wild-type p53 (*TP53^Wt^*) might have defects in other parts of the pathway [1]. In sarcomas, malignant tumours resembling mesenchymal tissue, amplification of *MDM2 *(murine double minute 2) is relatively common (20%) in tumours having *TP53^Wt^*, resulting in disabled p53 function because overexpressed MDM2 protein binds to and inactivates p53 [2,3]. Remaining tumours may have other aberrations in their p53 pathway, either p53 mutations (*TP53^Mut^*, 11-31% depending on subtype), or other changes in the downstream pathway that do not affect the level of MDM2 (*MDM2^Wt^*/*TP53^Wt^*, 11-88% depending on subtype) [4-6].

Sarcomas are among the more frequent cancers among children [7], and both children and adults are treated with intensive surgery, chemotherapy or radiation, or a combination of these. Currently used chemotherapy (e.g. Methotrexate, Cisplatin and Doxorubicin) is frequently inadequate, with 50-80% long-term survival depending on tumour subgroup [8,9] and associated with severe toxicity. Due to the frequent failure of prevailing therapy and unacceptable adverse effects there is an urgent need for new therapeutic modalities in sarcoma.

Nutlin-3a, a novel small-molecule inhibitor of the p53-MDM2 interaction, has been shown to be highly effective in killing osteosarcoma cells *in vitro *and reducing tumour burden *in vivo *[10,11]. Nutlin-3a displaces p53 from the binding pocket of MDM2 and thereby releases p53 from inhibition and proteasomal degradation, leading to induction of its downstream targets, cell cycle arrest, and apoptosis. Tumours with amplification of the *MDM2 *gene (*MDM2^Ampl^*) are most responsive to Nutlin, most likely due to otherwise intact downstream p53 signalling [11]. However, the apoptotic response in cancer cells with normal levels of MDM2 can vary dramatically, suggesting that other mechanisms or modifying factors are involved in the response to MDM2 antagonists. Several studies have suggested both p53-related and unrelated factors, such as caspases, BAX, PUMA, p73 and other apoptotic factors to be involved [10,12-14], and the status of the related *MDM4 *(also known as *MDMX*) gene has also been proposed to explain the responses to Nutlin [15-17].

Early studies suggest that MDM2 antagonists may be particularly effective in sarcomas because *MDM2 *is frequently amplified in these tumours [2,5,18,19]. We previously confirmed the effectiveness of Nutlin-3a as a single agent in *TP53^Wt^*/*MDM2^Ampl ^*liposarcomas [20]. However, since many sarcomas are *TP53^Wt ^*but do not have amplified *MDM2 *(are *MDM2^Wt^*), it would be of interest to investigate if Nutlin-3a could potentiate the response of *MDM2^Wt^*/*TP53^Wt ^*tumours to conventional chemotherapy. Since p53 mutations are very heterogeneous, different mutations sites could also imply different response to combined therapy. Nutlin has been shown to be synergistic with genotoxic drugs (e.g. Fludarabine, Chlorambucil, Doxorubicin, Etoposide, Melphalan and Cisplatin) in haematological malignancies, lymphoma, neuroblastoma and hepatocellular carcinoma, and with radiation in lung cancer [12,21-26], but antagonistic with antimitotic agents (e.g. Paclitaxel) in colon cancer cell lines [27]. Paclitaxel was shown to be synergistic in rhabdomyosarcoma cell lines [28]. Here, we investigate the effect of Nutlin-3a on sarcoma cell lines in combination with the current standard of therapy (e.g. Doxorubicin, Cisplatin and Methotrexate). These are well known and frequently used genotoxic drugs that induce cell cycle arrest and apoptosis through both p53-dependent and-independent mechanisms [29-31]. The latter group is represented by Methotrexate, which primarily inhibits dihydrofolate reductase (DHFR), but also glycinamide ribonucleotide formyltransferase (GARFT) and thymidylate synthetase (TS), all key components of nucleotide biosynthesis. Our studies show significant potentiation and/or reduction of effective dose of cytotoxic drugs by Nutlin, in both wild-type and mutated *TP53 *tumours, suggesting that clinical combination studies in sarcoma are warranted.

## Methods

### Cell lines and culture conditions

We selected 5 different sarcoma cell lines, three with *TP53^Wt ^*and two with *TP53^Mut^*. Two cell lines had *MDM2^Ampl ^*and three had *MDM2^Wt^*(Table 1). The cell line T778 (94778), kindly provided by Dr Florence Pedeutour, was established from a relapsed liposarcoma at Hopital de l'Arche [32]. Dr. A Thomas Look at St Jude's hospital, Memphis, USA, kindly donated the cell lines RMS13 [33] and OSA (available from the ATCC, Rockville, MD as SJSA-1 or CRL2098). SaOS-2 (HTB85) and U2OS (HTB96) were purchased from the ATCC. All cell lines were grown in RPMI 1640 medium (Bio Whittaker, Verviers Belgium) containing 20 mM HEPES and 2 mM GlutaMax (GIBCO BRL Life Technologies, Paisley UK), 50 IE/ml penicillin, 0,1 μg/ml streptomycin (Bio Whittaker, Verviers Belgium) and 8% heat-inactivated fetal bovine serum (PAA Laboratories, Pasching, Austria) in an environment containing 5% CO_2 _at 37°C.

**Table 1:** Cell line characteristics

Cell line	Histology	Patient sex/age	Origin	Site	TP53 status	MDM2 copy number	MDM2 mRNA level
T778	DDLS	F/69	Relapse	Retroperitoneum	wt	59.8 ± 1.8	28.5 ± 1.6

OSA (SJSA-1)	OS	M/19	Primary	Femur	wt	49.1 ± 1.1	43.2 ± 9.4

U2OS	OS	F/15	Primary	Tibia	wt	0.7 ± 0.1	3.1 ± 0.2

RMS13	RMS	M/17	Primary	Bone marrow	mut (exon 8)	1.9 ± 0.2	0.9 ± 0

SaOS-2	OS	F/11	Unknown	Unknown	del	1.4 ± 0.2	2.5 ± 0.2

Panel of cell lines tested. DDLS refers to de-differentiated liposarcoma, OS osteosarcoma and RMS rhabdomyosarcoma. RMS13 was initially classified *TP53^Wt^*, but reclassified as *TP53^Mut ^*after extended DNA sequence analysis. MDM2 copy number and expression were determined previously as described in Müller et al, 2007.

### Direct sequencing mutation analysis of *TP53*

Genomic DNA was extracted from cell lines using the standard phenol chloroform method. The total protein coding region of *TP53 *(exon 2 to 11) was amplified in five distinct fragments by using flanking intronic primers with M13 tails [34], generating products of 837, 464, 887, 300 and 275 base pairs respectively. In short, all the five fragments were amplified in separate PCR reactions using identical conditions for each primer pair. We used HotStar Polymerase (Qiagen, GmbH, Germany) for PCR. The quality and quantity of the resulting PCR product was evaluated on a polyacrylamide gel, and the product was purified using ExoSAP-IT (USB Corporation, Cleveland, Ohio, USA). Both 5' and 3' sequencing reactions were performed using BigDye terminator v3.1 kit from Applied Biosystems (Foster City, California, USA) and adding M13 primers to the PCR product. The resulting sequence product was further purified using Millipore multiscreen plates (Millipore, Billerica, Massachusetts, USA) with Sephadex G-50 Superfine (GE Healthcare, Chalfont St. Giles, United Kingdom), and subjected to sequencing on a 3730 DNA Analyzer (Applied Biosystems). Where a mutation was detected, a new independent PCR product was subjected to sequencing to confirm our findings.

### Drug sensitivity assay

Cellular integrity was measured as total cellular protein by the Sulforhodamine B (SRB) assay, which was performed essentially as described previously [35]. Briefly, in a 96-well plate (Becton Dickinson), cells were seeded in 100 μl/well at appropriate cell densities; 500 cells per well for OSA, 1 500 for T778, 10 000 for RMS13, 2 000 for U2OS and 8 000 for SaOS-2. At these densities the untreated control cells were growing exponentially during the entire incubation period. After 24 hours, 100 μl drug-containing medium was added and the cells were cultured for another 120 hours.

All cell lines were exposed to the drugs alone or in combination. The IC50 of the individual drug was determined where after combinations were made based on the ratio of the IC50's. Drugs were added in a constant ratio design. For Doxorubicin and Cisplatin three fixed ratios were set: Ratio 1:1 = IC50_Nutlin-3a_: IC50_x_, ratio 1:2 = IC50_Nutlin-3a_: 2 times IC50_x _and ratio 2:1 = 2 times IC50_Nutlin-3a: _IC50_x _, where x is the chemotherapy. For Methotrexate we used the 1:1 ratio only. When the ratio was set, a mixture of the two drugs was made and serially diluted to obtain a good dosage range.

After exposure, cells were fixed with 50% Trichloroacetic acid (TCA, Sigma-Aldrich, 25 μl/well), washed and stained with 0.4% Sulforhodamine B (SRB, Sigma-Aldrich) dissolved in 1% acetic acid (Merck, 50 μl/well), subsequently washed five times with 1% acetic acid to remove unbound stain. Protein bound stain was dissolved in 150 μl 10 mM Tris base (Merck). Optical density was measured at 540 nm in a Victor Wallac 1420 multi-label counter (Perkin Elmer, MA USA). Relative Growth was calculated as follows:

when OD_treated _- OD_zero _> 0; Cell death occurred if OD_treated _- OD_control _< 0, where OD_treated _represents the optical density of treated cells at the day of the assay, OD_control _the optical density of the untreated control cells and OD_zero _the optical density at the moment of drug addition. From the relative growth we obtain the fraction affected (Fa) as follows:

### Combination analysis

To evaluate the pharmacological interaction of the different combinations of drug treatments, we followed the method proposed by Chou et al. [36] Briefly, synergism, additivity or antagonism for the different combinations was calculated on the basis of the multiple drug-effect equation and quantitated by the combination index (CI) where CI = 1 indicates that the two drugs have additive effect, CI < 1 more than additive effect ("synergism") and CI > 1 less than additive effect ("antagonism"). The involved drugs were assumed mutually non-exclusive meaning that they have totally independent modes of action and the CI was calculated using the CalcuSyn software based on:

where (D_x_)_1 _and (D_x_)_2 _are the doses of drug 1 and drug 2 alone inhibiting x % whereas (D_1_) is the dose of drug 1 in combination with drug 2 and (D_2_) the dose of drug 2 in combination with drug 1 that gives the experimentally observed x inhibition. Since our aim was to achieve maximal effect of the drugs tested on cancer cells, a mean CI was calculated from data points with fraction affected (Fa) > 0.5. (Fa) < 0.5 would imply lower growth inhibition and a large fraction of the cell population would still grow. Fa < 0.5 was therefore considered not relevant. Furthermore we evaluated how much each drug dose in a synergistic combination could be reduced at a given effect level compared with the doses for each drug alone, the so-called dose-reduction index (DRI):

Notably, non-synergistic combinations could also result in reduced drug doses (DRI > 1) as illustrated by the example: if drug A and drug B each inhibit 50% and if (0.5A+ 0.5B) also inhibits 50% and both drugs have no overlapping toxicity toward the host, DRI ≥ 1 may still be beneficial [37].

### Immunoblotting

In the drug sensitivity assay we chose a constant ratio design with three fixed ratios as described above. By only including the 1:1 ratio in the western analysis, our intent was to eliminate the potential disturbing effect of different drug concentrations on the molecular mechanisms. Cells were treated for 24 hours with Nutlin-3a, Doxorubicin, Cisplatin, Methotrexate or described drug combinations. They were lysed in lysis buffer (supplemented with phosphatase and protease inhibitor cocktails (Sigma-Aldrich)) and sonicated. 15-30 μg of protein lysate was separated on 4-12% NuPage BIS-Tris gradient gels (Invitrogen, CA USA) and transferred to 0.45 μm PVDF membranes (Millipore Corp., MA USA) in blotting buffer with 20% (v/v) methanol using wet blot equipment from BIO-RAD, CA USA. Antibodies used (monoclonal except when noted): anti-p53 (1:3000 Santa Cruz sc-6243), anti-MDM2 (1:300, Chemicon MAB1434), anti-MDM4 (1:20000 Bethyl Labs), anti-PUMA (1:5000, Abcam), anti-β-actin (1:4000, Sigma-Aldrich), and polyclonal anti-p21 (1:300, Santa Cruz sc-397). All HRP-conjugated secondary antibodies were from Dako, Denmark. Bands were visualized using SuperSignal West Dura ECL (# 34076, Pierce, IL USA) and Kodak X-VIS film and software (Eastman Kodak, NY USA). Pictures were merged and presented using Photoshop (Adobe Systems, USA).

### Statistics

The Statistical package SPSS 13.0 was used. 2-tailed unpaired Student's T-test on transformed results was used for statistical analysis. Changes were considered to be significant when *P *< 0.05.

## Results

We investigated the effect of combining Nutlin-3a with the current standard of sarcoma therapy (Doxorubicin, Cisplatin, Methotrexate) on the growth of five sarcoma cell lines. The characteristics and sources of the cell lines used are described in Table 1. From our previous results we knew that the liposarcoma cell line T778 was sensitive to Nutlin-3a and we included the osteosarcoma cell line OSA with similar characteristics (*MDM2^Ampl^/TP53^Wt^*) as a control cell line. As representative of *MDM2^Wt^/TP53^Wt^*, we chose the osteosarcoma cell line U2OS. Finally, SaOS-2 and RMS13 were chosen as examples of two different cell lines with mutated p53. SaOS-2 is known to be *TP53^Null^*[38]. RMS13, previously described as *TP53^Wt^*, did not respond to Nutlin-3a, and was found to have a transversion in codon 280, exon 8, changing the amino acid from arginine to serine in the resulting protein. Protein analysis uncovered high levels of probably dysfunctional p53 protein and constitutively high p21 and PUMA (see below). This is consistent with a report that the Rh30 cell line, established independently from a xenograft from the same patient, also had this mutation [39] and illustrates the usefulness of Nutlin as a probe for p53 status. The other cell lines with *TP53^Wt ^*were validated by sequencing.

### Single drug treatment

**Figure 1:**
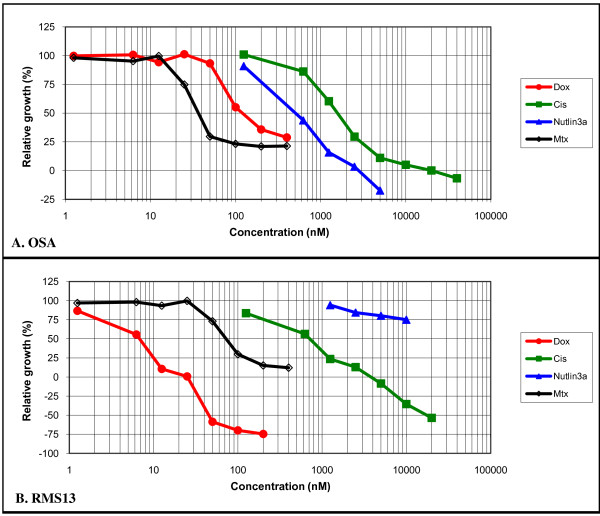
**Representative growth inhibition curves for OSA (A) and RMS13 (B) after Nutlin-3a, Doxorubicin, Cisplatin and Methotrexate exposures**. Exponentially growing cancer cells with *TP53^Wt^*(OSA) or *TP53^Mut^*(RMS13) were incubated with a range of drug concentrations for 120 h. Relative growth was measured by the SRB assay.

The effect of each drug alone is presented in Table 2. The *MDM2^Ampl ^*cell lines (OSA and T778) were sensitive to Nutlin-3a and showed clear dose dependence, whereas the *MDM2^Wt^/TP53^Wt ^*U2OS was less sensitive, with an IC50 about twice that for the *MDM2^Ampl ^*cell lines. Consistent with previous results, we were not able to reach 50% growth inhibition for the *TP53^Mut ^*cell lines (SaOS-2 and RMS13) when using Nutlin-3a alone. Cisplatin, Doxorubicin and Methotrexate also inhibited cell growth in a concentration-dependent manner. Figure 1 shows representative growth inhibition curves for the *MDM2^Ampl^*/*TP53^Wt ^*OSA and *MDM2^Wt^*/*TP53^Mut ^*RMS13 exposed to single drugs. In all cell lines tested, Doxorubicin and Methotrexate were more cytotoxic than Cisplatin or Nutlin-3a when used alone (Table 2), and Methotrexate was most effective in the *MDM2^Ampl ^*cell lines. Doxorubicin on the other hand was most cytotoxic in the *TP53^Mut ^*line, RMS13. For Cisplatin, the most sensitive cell line tested was OSA, followed by RMS13, U2OS, T778 and SaOS-2.

**Table 2:** Growth inhibition by Nutlin-3a, Doxorubicin, Cisplatin and Methotrexate for the sarcoma cell lines

Mean IC50 ± SE(nM)					
**Drugs**	**OSA**	**T778**	**U2OS**	**RMS13**	**SaOS-2**

Nutlin-3a	527 ± 131	658 ± 138	1024 ± 485	na	na
Dox	62 ± 77	62 ± 31	40 ± 35	14 ± 4	77 ± 12
Cis	758 ± 592	1566 ± 1369	1037 ± 596	855 ± 143	4828 ± 4855
Mtx	30 ± 9	59 ± 74	181 ± 153	106 ± 34	636 ± 806

Cells were exposed to a concentration range of Nutlin-3a, Doxorubicin, Cisplatin and Methotrexate for 120 h. Values (IC50 in nM) are means ± standard error of the mean (SE) for 3-5 experiments. RMS13 and SaOS-2 were insensitive to Nutlin-3a consistent with their *TP53^Mut^*.

### Combination treatments

**Figure 2:**
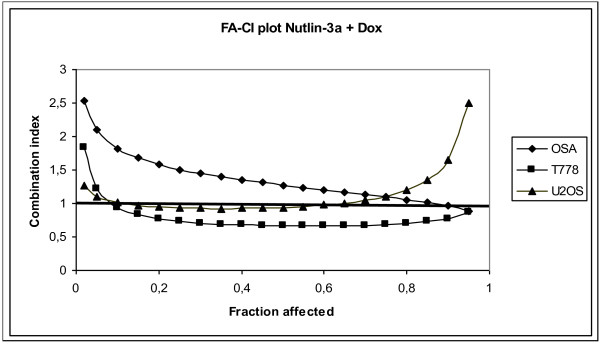
**Illustrative Fa-CI plot for the combination of Nutlin-3a and Doxorubicin using a 1:1 fixed drug ratio**. CI values are calculated from each Fa for the three Nutlin-3a sensitive cell lines in the study. Here we demonstrate average synergism at Fa > 0.5 for T778 whereas OSA and U2OS display average antagonism.

Multiple drug-effect analysis was performed for combinations of Nutlin-3a with Doxorubicin, Cisplatin or Methotrexate using the CalcuSyn software. Fraction affected (Fa) values (indicating the fraction of cells inhibited after drug exposure) were obtained after exposure of the cells to a series of drug concentrations. To indicate the effects at different Fa values, combination index (CI) values were calculated for each Fa. For the *TP53^Mut ^*cell lines RMS13 and SaOS-2 we were not able to calculate any combination effect since the cell lines did not respond to Nutlin-3a. Figure 2 shows an Fa-CI plot illustrating the effects of Nutlin-3a and Doxorubicin in a 1:1 fixed drug ratio combination and demonstrates synergism at Fa > 0.5 for T778 whereas on average the other two cell lines display less than additive effects. As we wanted to determine the combination effect at concentrations showing efficacy, we limited the analysis to Fa greater than 0.5. The mean CI values of the various combinations for Fa > 0.5 are shown in Table 3A. Overall we found additive or more than additive effects when Nutlin-3a was combined with Doxorubicin for 4 out of 6 ratios tested for *MDM2^Ampl ^*cell lines, whereas Cisplatin in combination with Nutlin-3a showed less than additive effect for 5 out of 6 ratios. Nutlin-3a together with Cisplatin or Doxorubicin also showed less than additive effects in the *MDM2^Wt ^*cell line U2OS. Methotrexate on the other hand showed more than additive effect when combined with Nutlin-3a for T778 and U2OS, *MDM2^Ampl ^*and *MDM2^Wt^*, respectively.

**Table 3:** Effects of combining Nutlin-3a with chemotherapy

A							
**Combination Index**							

**Drug combination with Nutlin-3a**		**OSA**		**T778**		**U2OS**	
		**CI ± SE**		**CI ± SE**		**CI ± SE**	

**Dox**	1:1	1,2 ± 0,3	antagonism	0,7 ± 0,2	synergy	1,1 ± 0,2	antagonism
	1:2	1,0 ± 0,1	additivity	1,4 ± 0,4	antagonism	2,1 ± 0,7	antagonism
	2:1	0,8 ± 0,1	synergy	0,9 ± 0,2	synergy	1,7 ± 0,6	antagonism
**Cis**	1:1	1,9 ± 0,6	antagonism	1,2 ± 0,2	antagonism	1,4 ± 0,3	antagonism
	1:2	2,1 ± 0,6	antagonism	1,2 ± 0,4	antagonism	2,4 ± 0,6	antagonism
	2:1	1,1 ± 0,3	antagonism	0,7 ± 0,2	synergy	1,4 ± 0,2	antagonism
**Mtx**	1:1	1,4 ± 0,4	antagonism	0,9 ± 0,4	synergy	0,9 ± 0,3	synergy

**B**							

**Dose Reduction index values (mean ± s.e.)**							

		**75%**		**90%**		**95%**	
**Drug combination**		**Nutlin-3a**	**Chemotherapy**	**Nutlin-3a**	**Chemotherapy**	**Nutlin-3a**	**Chemotherapy**

**OSA**	Nutlin-3a+Dox 1:1	1.5 ± 0.4	6.8 ± 3	1.4 ± 0.4	6.8 ± 2	1.3 ± 0.4	7.2 ± 2
	Nutlin-3a+Dox 1:2	2.6 ± 0.5	2.5 ± 0.6	2.5 ± 0.5	3.2 ± 0.5	2.5 ± 0.6	3.8 ± 0.7
	Nutlin-3a+Dox 2:1	1.9 ± 0.3	7.6 ± 2	1.4 ± 0.1	7.8 ± 2	1.2 ± 0.0	8 ± 2
	Nutlin-3a+Cis 1:1	2.0 ± 0.5	2.0 ± 0.4	1.6 ± 0.4	1.9 ± 0.5	1.4 ± 0.4	1.9 ± 0.6
	Nutlin-3a+Cis 1:2	2.6 ± 0.7	1.3 ± 0.3	2.1 ± 0.5	1.2 ± 0.3	1.8 ± 0.5	1.2 ± 0.3
	Nutlin-3a+Cis 2:1	3.5 ± 2	6.3 ± 2	2.4 ± 1	4.7 ± 1	1.8 ± 0.8	4.1 ± 1
	Nutlin-3a+Mtx 1:1	1.0 ± 0.1	3.7 ± 0.9	0.7 ± 0.2	4.7 ± 0.6	0.7 ± 0.3	5.7 ± 1
							
**T778**	Nutlin-3a+Dox 1:1	2.2 ± 0.5	8.1 ± 2	2.1 ± 0.6	14.1 ± 6	2.1 ± 0.7	21.1 ± 9
	Nutlin-3a+Dox 1:2	1.7 ± 0.4	3.4 ± 1	1.2 ± 0.3	3.7 ± 1	1.0 ± 0.2	4.0 ± 1
	Nutlin-3a+Dox 2:1	1.2 ± 0.2	9.2 ± 3	0.8 ± 0.2	9.3 ± 3	0.6 ± 0.2	9.5 ± 3
	Nutlin-3a+Cis 1:1	2.1 ± 0.2	2.3 ± 0.4	1.8 ± 0.2	2.9 ± 0.9	1.6 ± 0.2	3.7 ± 2
	Nutlin-3a+Cis 1:2	3.7 ± 1.2	2.0 ± 0.6	3.4 ± 1	2.9 ± 2	3.3 ± 1	4.1 ± 3
	Nutlin-3a+Cis 2:1	3.2 ± 1	6.8 ± 2	2.8 ± 1	9.9 ± 6	2.5 ± 1	13.5 ± 9
	Nutlin-3a+Mtx 1:1	5.2 ± 2	3.1 ± 1	3.9 ± 2	2.3 ± 0.9	3.5 ± 2	1.8 ± 0.8
							
**U2OS**	Nutlin-3a+Dox 1:1	1.2 ± 0.2	9.5 ± 4	1.1 ± 0.4	6.0 ± 2	1.2 ± 0.5	4.8 ± 2
	Nutlin-3a+Dox 1:2	1.2 ± 0.4	4.2 ± 2	1.3 ± 0.4	4.2 ± 2	1.3 ± 0.4	4.2 ± 2
	Nutlin-3a+Dox 2:1	1.1 ± 0.4	13.1 ± 5	1.1 ± 0.5	10.6 ± 4	1.2 ± 0.5	9.3 ± 3
	Nutlin-3a+Cis 1:1	1.8 ± 0.5	3.0 ± 0.7	1.9 ± 1	2.6 ± 1	2.1 ± 1	2.8 ± 2
	Nutlin-3a+Cis 1:2	1.6 ± 0.4	1.4 ± 0.4	1.4 ± 0.6	1.0 ± 0.3	1.4 ± 0.7	0.9 ± 0.4
	Nutlin-3a+Cis 2:1	1.4 ± 0.2	6.1 ± 3	1.1 ± 0.2	4.1 ± 0.8	0.9 ± 0.2	3.5 ± 0.7
	Nutlin-3a+Mtx 1:1	1.5 ± 0.4	6.9 ± 2	1.3 ± 0.4	6.2 ± 2	1.1 ± 0.4	5.8 ± 2

**A **Combination Index (CI) values listed in the table are means ± standard error of the mean (SE) for Fa-values > 0.5, indicating additivity, more than additivity (synergy) or less than additivity (antagonism). Values were determined experimentally and calculated using the Chou and Talalay method and CalcuSyn software. Relative growth was assessed 120 h after drug treatment. Data is shown as combinations of Nutlin-3a with Doxorubicin, Cisplatin or Methotrexate in 1:1, 1:2 and 2:1 ratios. Since Nutlin-3a is not active in RMS13 and SaOS-2 it was by definition not possible to calculate their CI. **B **Dose Reduction Index (DRI) values for the three combinations at 75, 90 and 95% levels of growth inhibition of OSA, T778 and U2OS cell growth.

CI < 1: more than additivity (synergy)

CI = 1: additivity

CI > 1: less than additivity (antagonism)

As expected, synergism, corresponding to CI < 1, always yielded a favourable dose reduction index (DRI > 1) for both drugs. The DRI values at IC_75_, IC_90 _and IC_95 _are reported in Table 3B. These indicate that chemotherapy doses may be significantly reduced for combinations with Nutlin-3a that are synergistic. In both *MDM2^Ampl ^*cell lines, Doxorubicin could be reduced 10-fold and in T778 the Cisplatin dose could also be significantly reduced when combined with Nutlin at a 2:1 ratio. In addition, in cell lines being *MDM2^Ampl ^*or *MDM2^Wt^*, T778 and U2OS respectively, we found that reduced dose of Methotrexate would suffice when combined with Nutlin-3a. Some combinations that were not synergistic also maintained efficacy with reduced level of standard cytotoxic drugs (DRI > 1) when combined with Nutlin-3a. This holds for all combinations with Doxorubicin or Methotrexate as well as some with Cisplatin. Moreover, as depicted in Table 4 Nutlin-3a clearly potentiated Doxorubicin and Cisplatin in RMS13 and SaOS-2 whereas Methotrexate was inhibited.

**Table 4:** Growth inhibition of combination treatment in *TP53^Mut ^*cell lines

Mean IC50 ± SE(nM)						
	**Doxorubicin**		**Cisplatin**		**Methotrexate**	
	**alone**	**combination**	**alone**	**combination**	**alone**	**combination**

**RMS13**	14 ± 4	6 ± 3	855 ± 143	244 ± 85	106 ± 34	253 ± 75
**SaOS-2**	77 ± 12	40 ± 17	4828 ± 4855	517 ± 229	636 ± 806	na

RMS13 and SaOS-2 cells were exposed to a concentration range of Nutlin-3a, Doxorubicin, Cisplatin, Methotrexate and combinations for 120 h. Values (IC50 in nM) are means ± standard error of the mean (SE) for 3-5 experiments. The decrease observed in the combination of Nutlin-3a with Doxorubicin or Cisplatin, indicates that Nutlin-3a potentiates Doxorubicin and Cisplatin, whereas the increase in IC50 observed for the combination with Methotrexate indicates that Nutlin-3a inhibits Methotrexate.

### Protein analysis

**Figure 3:**
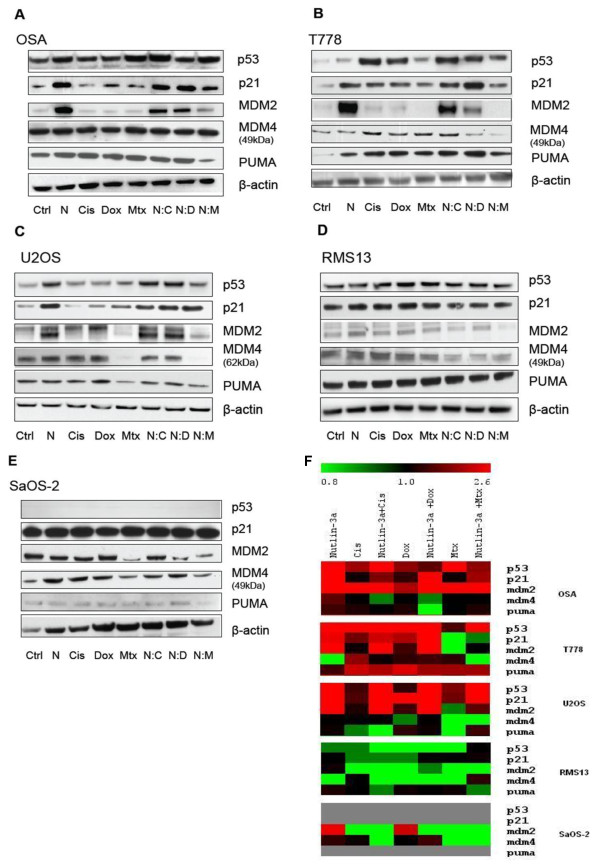
**Nutlin-3a modulates MDM2-binding proteins in a p53-dependent manner**. Cell lines were exposed to drug-free media (Ctrl), Nutlin-3a (N), Cisplatin (Cis), Doxorubicin (Dox), Methotrexate (Mtx), Nutlin-3a combined with Cisplatin (N:C), Doxorubicin (N:D) or Methotrexate (N:M) in 1:1 ratios. **A **and **B **illustrate the induction of p53 pathway components by N:C and N:D combinations in the two *MDM2^Ampl ^*cell lines OSA and T778. **C **U2OS (*MDM2^Wt^*) displays specific reduction in MDM4 (62kDa) after Methotrexate exposure alone or in combination with Nutlin-3a. **D **and **E **display the *TP53^Mut ^*cell lines RMS13 and SaOS-2 respectively. The figure shows representative western blots of 3 independent biological experiments, targeting p53, p21, MDM2, MDM4, PUMA, and β-actin (loading control). Figure **F **illustrates quantitative measurements of the same three western blot experiments, values representing mean fold induction ranging from 0.8 (green) to 2.6 fold (red).

Since most classic cytotoxic drugs exert their antitumour effect at least in part by activation of the p53 pathway, we investigated some key downstream p53 targets by Western blotting. As expected, Nutlin-3a induced accumulation of p53 protein in the *MDM2^Ampl ^*cell lines (Figure 3A-B and 3F), followed by activation and accumulation of p53 targets p21 and MDM2. Although the response was weaker than for Nutlin-3a alone, Doxorubicin and Cisplatin also induced p53 and its downstream targets as did the combinations. Similar to Nutlin-3a, Methotrexate induced and increased p53 in these cells, but downstream targets such as p21 and MDM2 appeared unaffected in T778. PUMA was unaffected in OSA, but induced to a similar level by all treatments in T778. The *MDM2^Wt ^*cell line U2OS (Figure 3C and 3F) also displayed significant induction of p53, p21, and MDM2 after Nutlin-3a treatment alone and in combinations, whereas Cisplatin, Doxorubicin and Methotrexate alone had less effect. In this cell line, neither Nutlin-3a nor the classical drugs affected the level of PUMA, but MDM4 was completely knocked down by Methotrexate (Figure 3C and 3F), both alone and in combination with Nutlin-3a. It should be noted that U2OS expressed a 62 kDa variant of MDM4, whereas all the other lines expressed the 49 kDa band (Figure 3). In cell lines where p53 was not functional (RMS13 and SaOS-2), the downstream targets were not affected by any of the treatments, as expected (Figure 3D-F). Taken together we found that the combination treatments induced the p53 pathway proteins examined in all *TP53^Wt ^*cell lines, whereas in *TP53^Mut ^*cell lines they were unaffected (Figure 3A-F).

## Discussion

Current treatment options for sarcomas, based on decades of optimising high dose chemotherapy, surgery and radiation, have reached a plateau with long-term survival limited to 50-80% depending on tumour subgroup [8,9]. Although some new targeted treatments have been successful in other cancer types and also in some sarcoma subtypes (e.g. GIST) [40], these treatments rarely increase long-term survival. Since sarcomas are among the most frequent childhood cancers, the possibility of reducing the long-term adverse effects of high-dose chemotherapy is an important objective. Thus, new treatments targeting key cancer pathways are highly desirable. One such pathway is the p53 tumour suppressor pathway.

Although *TP53 *mutations are not so frequent in sarcomas [41], the pathway may also be inactivated by amplification of the *MDM2 *gene [2,5,18] which is observed almost exclusively in tumours where *TP53 *is wild-type. However, these two mechanisms do not account for all tumours, and although some complementary mechanisms have been suggested [6], it is still unclear if and how the pathway is inactivated in the remainder of sarcomas. If tumours contain defects of the p53 pathway that are upstream of p53 or suppress p53 levels, activation of p53 could bypass the defect and, provided the right signals are present, either induce apoptosis directly or sensitize the cells to induction of apoptosis by traditional therapy.

Small-molecule MDM2 antagonists such as Nutlin-3a are interesting in this aspect not only as therapeutic agents, but as tools to study p53 pathway functionality [11]. Nutlins efficiently activate p53 signalling and induce cell cycle arrest and apoptosis in sensitive cancer cells, while sparing most normal cells when tested in vitro [10-13,15,23,27,42]. However, their safety in humans is still unknown, especially when combined with traditional cytotoxic therapy. Although it has been shown that Nutlin alone is very efficacious in *MDM2^Ampl ^*sarcoma cell lines *in vitro *and *in vivo *[11,20], the effects of combinations with standard therapeutics in sarcomas is still unknown. The aim of this study was to investigate possible synergistic interactions between traditional sarcoma drugs and Nutlin-3a.

As expected, cell lines with wild-type *TP53 *responded to Nutlin-3a exposure by activation of p53 and its downstream targets, including p21 and MDM2, whereas tumours with mutated *TP53*, irrespective of mutation site, did not respond to Nutlin-3a alone. *MDM2^Ampl ^*cell lines were more sensitive to Nutlin-3a compared to the *MDM2^Wt ^*cell lines in spite of similar levels of induction of p21, MDM2 and PUMA. This is probably the result of other yet undiscovered defects in the downstream p53 signalling in the *MDM2^Wt ^*cells. MDM4 is an MDM2 homologue that shares some of the same p53 binding sites as MDM2, although there are some differences [43]. It has been suggested that high levels of MDM4 may counteract Nutlin because its binding to p53 is not inhibited by the treatment [15,16,44], although the hypothesis is still controversial [45].

The genotoxic agents tested in this study are commonly used in sarcoma treatment. Doxorubicin and Methotrexate were quite effective in these cell lines, whereas higher doses of Cisplatin and Nutlin-3a were required in the *TP53^Wt ^*lines. Neither Doxorubicin nor Cisplatin showed consistent differences in effect in relation to *MDM2 *amplification or *TP53 *mutation, but the *TP53 *mutated cell line (RMS13) was more sensitive to all three genotoxic agents tested than the *TP53 *null cell line (SaOS-2). Methotrexate was more effective in the lines with *MDM2 *amplification, suggesting some relation between MDM2 and the Methotrexate mechanism of action that warrants further investigation.

The DRI values clearly illustrated the benefit of the synergistic combinations, with 10-fold reductions in Doxorubicin and Cisplatin when combined with Nutlin-3a, but also combinations with less than additive effects showed promising DRI values. Nutlin-3a and Doxorubicin or Cisplatin were less than additive in the *MDM2*^Wt^/*TP53^Wt ^*U2OS cell line. This is consistent with previous results in colon (HCT116) and ovarian (A2780) cancer cells, where Nutlin was cytoprotective in combination with Cisplatin [14]. Compellingly the *TP53^Mut ^*cell lines, RMS13 and SaOS-2, showed a significant sensitization towards Cisplatin and Doxorubicin at IC50 when combined with Nutlin-3a. This apparently p53-independent effect of Nutlin-3a is in line with previous publications [46-48], and suggests efficacy against a wide spectrum of tumours. p73 has been proposed to be one of the factors responsible for the effect observed in *TP53*-null or-mutant cells. It was reported that when cells with dysfunctional *TP53 *were exposed to high concentrations of Nutlin (e.g. 20-30 μM), p73 was displaced from its binding to MDM2, resulting in cell cycle arrest and apoptosis [25,46,49]. Nutlin combined with Cisplatin or Doxorubicin has also previously been reported to induce apoptosis much more efficiently than either drug alone in cells with dysfunctional *TP53*, most likely due to E2F1-mediated up-regulation of p73 activity [48].

The third chemotherapy tested here, Methotrexate, acts through nucleotide metabolism, for which E2F1 is a central transcription factor [50,51]. Additional targeting of the p53 pathway by Nutlin-3a could be complementary to this mode of action [52]. We found synergistic effects for both *TP53^Wt^*/*MDM2*^Ampl ^and *TP53^Wt^*/*MDM2*^Wt ^cell lines and DRI values that implied that Methotrexate could be reduced ten-fold when combined with Nutlin-3a. In the *MDM2*^Wt ^cell line U2OS, treatment with Methotrexate decreased MDM4 to undetectable levels both alone and in combination with Nutlin-3a. According to the current hypothesis, reduction of MDM4 could lead to more unbound p53 [53], but since this reduction also happened with Methotrexate only, it would not contribute to the observed synergy. On the other hand, Nutlin-3a can indirectly reduce the level of E2F1 through MDM2 mediated degradation [48], and recent results also document the reduction of DHFR through MDM2 [54]. It is tempting therefore to speculate on a double inhibition of the nucleotide metabolism when using Methotrexate together with Nutlin-3a. The inhibitory effect observed in the *TP53^Mut ^*cell line (RMS13), could not be explained by this and clearly needs further investigation.

## Conclusions

Our in vitro data suggest that MDM2 antagonists could offer effective therapy for sarcoma either as single agent or in combination with prevailing chemotherapy, and may significantly reduce the genotoxic burden with the same or better antitumour effect. Further work with animal models of sarcoma and ultimately clinical studies needs to be done to evaluate and optimize combinations of Nutlin and current chemotherapies. Since clear biomarkers are still lacking, clinical trials should be followed up with detailed tumour profiling.

## Competing Interests

Lyubomir Vassilev is employed by Roche, the company which developed Nutlins, and as such could be perceived to have a vested interest in these results. This has however had no consequences for how the studies were performed and the results interpreted.

## Authors' Contributions

HOO carried out the drug sensitivity assays and the combination analysis, performed the statistical analysis and drafted the manuscript. EBP carried out the immunoassays. PN participated in the design of the study and drug sensitivity assays. MB and RAL carried out the sequence analysis. LTV participated in the study design and helped to draft the manuscript. OM conceived the study, participated in its design and coordinated and helped to draft the manuscript. All authors read and approved the final manuscript.

## Pre-publication history

The pre-publication history for this paper can be accessed here:

http://www.biomedcentral.com/1471-2407/11/211/prepub
